# Determination of Total Flavonoids Contents and Antioxidant Activity of* Ginkgo biloba* Leaf by Near-Infrared Reflectance Method

**DOI:** 10.1155/2018/8195784

**Published:** 2018-08-01

**Authors:** Ling-jia Zhao, Wei Liu, Su-hui Xiong, Jie Tang, Zhao-huan Lou, Ming-xia Xie, Bo-hou Xia, Li-mei Lin, Duan-fang Liao

**Affiliations:** ^1^College of Pharmacy, Hunan University of Chinese Medicine, Changsha 410208, China; ^2^Key Laboratory for Quality Evaluation of Bulk Herbs of Hunan Province, Hunan University of Chinese Medicine, Changsha 410208, China; ^3^School of Management of Information Engineering, Hunan University of Chinese Medicine, Changsha 410208, China; ^4^Institute of Material Medical, Zhejiang Chinese Medical University, Hangzhou, Zhejiang, China

## Abstract

**Background:**

Total flavonoids content (TFC) is one of the most important quality indexes of* Ginkgo biloba* leaf, and it is concerned with total antioxidant activity. Near-infrared spectroscopy (NIR) method has showed its advantages in fast, accurate, qualitative, and quantitative analysis of various components in many quality control researches. In this study, a calibration model was built by partial least squares regression (PLSR) coupling with NIR spectrum to quantitatively analyze the TFC and total antioxidant activity of* Ginkgo biloba* leaf.

**Results:**

During the model establishing, some spectrum pretreatment and outlier diagnosis methods were optimized to establish the final model. The coefficients of determination (*R*^*2*^) for TFC and total antioxidant activity prediction were 0.8863 and 0.8486, respectively; and the root mean square errors of prediction (RMSEP) were 2.203 mg/g and 0.2211 mM/g, respectively.

**Conclusion:**

These results showed that NIR method combined with chemometrics is suitable for quantitative analysis of main components and their activities and might be applied to quality control of relevant products.

## 1. Introduction

*Ginkgo biloba *L. (Ginkgoaceae) is an ancient tree growing in China for thousands of years. In recent decades,* Ginkgo biloba *L. occupies a prominent position among the best-selling natural products owing to its reliable and remarkable biological activities [1, 2]. Some studies and clinical trials have observed that* Ginkgo biloba *L. shows potent actions on cardiovascular system and cerebral vascular activity. Furthermore, due to its antioxidant properties, it has been used in Alzheimer's patients [3–5].

The main active components in* Ginkgo *leaf are flavonoids and terpene trilactones. Because only several kinds of terpene trilactones were found in* Ginkgo*, the pharmacological effects of them are relatively clear and the corresponding quality evaluation is simply achieved [6, 7], while more than 70 kinds of flavonoids were identified, which associate with various kinds of pharmacological actives [8–11]. Therefore, more researches have been conducted which focused on flavonoids in Ginkgo as their broad-spectrum of antioxidant and free-radical scavenging activity. Thus, total flavonoids content is often considered as an important quality index of* Ginkgo biloba* leaf. Determination of total flavonoids contents in* Ginkgo biloba* leaf and further estimating their antioxidant are of great importance to their qualities [7, 12].

In recent years, flavonoids in* Ginkgo biloba* L. have received considerable attention in various literatures, especially due to their widely recognized free-radical scavenging activity. TFC is often considered an important quality index of* Ginkgo biloba* products and samples, while DPPH radical methods are a stable N-centered radical at room temperature that is widely employed to assess the radical scavenging properties of antioxidants, such as total flavonoids. The traditional methods for determining total flavonoids in botanical materials are based on chemical extraction and couple with various analytical techniques, such as HPLC [13–15], GC [16], and ultraviolet spectrometry [17]. These methods are precise, and some methods are used as the reference methods for TFC detection. Yet, these methods all needed the sample pretreat or extraction process, which are often time-consuming and destructive. Therefore, a rapid, accurate, and even nondestructive analytical method is needed to identify the TFC and further determine their actives for the quality control of* Ginkgo biloba *L.

Near-infrared spectroscopy (NIR) has been used for various applications, such as quality estimation and quality control of various food, agriculture, and pharmaceutical products. There are also many researches based on NIR in herbal quality control and main contents analysis, and they show the advantages, including simple sample preparation and rapid and simultaneous analysis of several analytes in a large number of samples. The NIR spectra combining with appropriate mathematical models and pattern recognition techniques can be used to qualitatively and quantitatively determine quality of various products [12, 18–20].

Some studies have been published by coupling near-infrared spectroscopy with chemometrics methods to qualitative and quantitative analysis of flavonoids concentrations in various botanical leaves and relevant products, including* Ginkgo* leaf. Shi et al. determined the TFC in fresh* Ginkgo* leaves with different colors by using the NIR spectroscopy; furthermore they also analyzed the basic structure of flavonoids and relationship of wavelength regions [12]. Liu et al. published their reviews about the roles of flavonoid and its broad-spectrum free-radical scavenging activities in* Ginkgo biloba* chemical analysis and quality control [8]. Geng et al. established a quantitative near-infrared diffuse reflectance spectroscopy method for the simultaneous determination of three flavonol aglycones in* Ginkgo biloba *extracts [21]. Yet, no research has been reported to quantitatively analyze the TFC and their total antioxidant activity in* Ginkgo biloba* samples, simultaneously.

Based on these reasons, we aimed to establish a calibration model to quantitatively analyze the TFC in Ginkgo* biloba* leaves and further quantitatively estimate their antioxidant properties. During the model establishing process, some NIR signal pretreat methods were adopted to optimize the calibration model. The feasibility of combining NIR spectroscopy with chemometrics methods to rapid and nondestructive determination of TFC and their antioxidant properties was investigated.

## 2. Material and Methods

### 2.1. Chemicals and Materials

1,1-Diphenyl-2-picrylhydrazyl (DPPH) and Trolox were purchased from Sigma-Aldrich Chemical Co. (St. Louis, MO, USA). NaNO2, NaOH, and Al (NO3)3 were analytical grade and acquired from Shanghai Macklin Biochemical Co., Ltd. (Shanghai, China). Rutin was obtained from the National Institution for Food and Drug Control (Beijing, China). Distilled water was filtered by using a Milli-Q water-purification system (Millipore, Bedford, MA, USA). 113 batches of samples were collected from Zhejiang Province. All samples were powdered by a grinder mill after dried and passed through 60-mesh place before analysis.

### 2.2. Total Flavonoids Content (TFC)

The concentrations of flavonoids were quantified based on a colorimetric assay method [12], with slight modifications. Briefly, rutin was used as a standard to establish calibration linear with function:

(1) A=8.0045  C+0.0914;r=0.9959r=linear range

0.90-1.00 g samples were weighted, and 10 mL 60% ethanol aqueous was used to extract flavonoids from theses samples with supersonic (KQ-300DE, Kunshan Ultrasonic Equipment Co., China) for 30 min. These samples were further centrifuged at 3000 *∗* g. All the supernatant was transferred to 25 mL volumetric flask and then was fixed to 25 mL with 60% ethanol aqueous. 1.5 mL of each extracts and 4.5 mL of distilled water were pipetted into a 25 mL tube and then mixed with 1 mL 5% (w v^−1^) NaNO_2_ solutions. After incubation for 6 min, 1 mL of the 10% (w v^−1^) Al (NO_3_)_3_ solutions was added to the mixture. The mixture was kept for 6 min before adding 10 mL 4% (w v^−1^) NaOH solutions and fixed to 25 mL with 60% ethanol aqueous. Finally, the mixture was reacted for 15 min and the absorbance of the mixture solution was measured with a spectrophotometer (SP-1901, Shanghai Spectrum Instruments Co., China) at 510 nm against a blank containing 5 mL of extraction solvent. Samples were independently analyzed in triplicate times, and the mean of three tests were used and the total flavonoid content was expressed as mg rutin equivalent per g dry weight (DW).

### 2.3. Determination of the Total Antioxidant Activity

DPPH radical scavenging activity was determined as described by Okawa et al. [22] with a slight modification. Solutions of known Trolox concentration were used for calibration. 2 *μ*L of samples or Trolox was mixed with 250 *μ*L of methanolic DPPH. The homogenate was shaken vigorously and kept in darkness for 30 min. Absorption of the samples was measured on the spectrophotometer at 515 nm. Results were expressed as Trolox equivalent per g of dry weight (mM TE g dried extract^−1^).

### 2.4. NIR Spectra Acquisition and Preprocessing

The NIR spectra were measured in a diffuse reflectance mode by Antaris II FT-NIR spectrophotometer (Thermo EIectron Co., USA) equipped with an integrating sphere. The spectra (4000 to 8000 cm^−1^ were analyzed, and total 4150 points/spectrum) were collected in the log (1/R) mode which was converted by the reflectance value (R). Each sample (0.5 g) was placed in the sample cup, each sample was measured three times, and the mean of the three spectra was used for further statistical analysis. The temperature was kept around at 25°C.

### 2.5. Multivariate Calibration Methods Establishing

The whole establishing process of calibration model has been described as follow steps.

Firstly, Kennard and Stone algorithm (K-S) [23] was adopted to split samples into calibration dataset (80%) and prediction dataset (20%), respectively. Then, the calibration dataset was used to develop calibration model for TFC and its antioxidant activity by using the partial least squares regression (PLSR). In calibration model, the number of PLS factors were optimized by 10-fold cross-validation method. It was performed as follows: (1) 90% of the calibration dataset samples were used to form the calibration model, and the remaining 10% samples were used to validate this model, and the procedure was repeated by 10 times; (2) the root mean square error of cross-validation (*RMSECV*) was then calculated as follows:

(2) $$\begin{array}{c} RMSECV=\sqrt{\frac{1}{n}\overset{n}{\underset{i=1}{\sum }}{\left({\hat{y}}_{i}-y_{i}\right)}^{2}} \end{array}$$

where n is the number of samples in the calibration set, *y*_*i*_ is the measured result for sample *i*, and ${\hat{y}}_{i}$ is the predicted value of sample *i*. The performances of the optimal model were evaluated according to root mean square error of calibration (*RMSEC*).* RMSEC* is calculated as follows:

(3) $$\begin{array}{c} RMSEC=\sqrt{\frac{1}{I_{c}}\overset{I_{c}}{\underset{i=1}{\sum }}{\left({\hat{y}}_{i}-y_{i}\right)}^{2}} \end{array}$$

where *I*_*c*_ is the number of samples in the calibration set, *y*_*i*_ is the measured result for sample*i*, and ${\hat{y}}_{i}$ is the predicted value of sample *i*.

Then, the optimal model which was validated by prediction samples in the prediction dataset* RMSEP* is calculated as follows:

(4) $$\begin{array}{c} RMSEP=\sqrt{\frac{1}{I_{p}}\overset{I_{p}}{\underset{i=1}{\sum }}{\left({\hat{y}}_{i}-y_{i}\right)}^{2}} \end{array}$$

where *I*_*p*_ is the number of samples in the prediction dataset, *y*_*i*_ is the measured result for sample *i*, and ${\hat{y}}_{i}$ is the predicted value of sample *i*.

Correction coefficient between the predicted value of PLSR model and the measurement value is calculated as follows for both the calibration and prediction set:

(5) $$\begin{array}{c} R=\sqrt{1-\frac{\sum_{i=1}^{n}{\left({\hat{y}}_{i}-y_{i}\right)}^{2}}{\sum_{i=1}^{n}{\left(y_{i}-\bar{y}\right)}^{2}}} \end{array}$$

where* n* is the number of samples in the calibration or the prediction set and $\bar{y}$ is the mean of measurement value for the calibration or the prediction set.

### 2.6. Spectral Signal Preprocessing

In the model establishing process, some signal pretreat need be optimized for achieving the best calibration model. In this study, some data preprocessing methods were used to process these NIR signals, such as multiplicative scattering correction (MSC), standard normal transformation (SNV), moving window smoothing, and Savitzky-Golay first derivative or second derivative (S/G 1st/2nd der). The detailed descriptions of these process methods can be found in previous researches [23–25]. All of the algorithms were implemented in MATLAB 8.0.1, and all the programs were written by own group (Mathworks).

## 3. Results and Discussion

### 3.1. TFC and Antioxidant Activity

The reduction capability of DPPH radical was determined by the decrease in absorbance by reduced DPPH to by plant antioxidants [26]. Therefore, getting systematic knowledge of flavonoids in* Ginkgo biloba* L. and their activities are highly important for the research and development of this plant. The results of TFC of these samples and their antioxidant activities were listed in Table 1.

### 3.2. Near-Infrared Spectra

**Figure 1:**
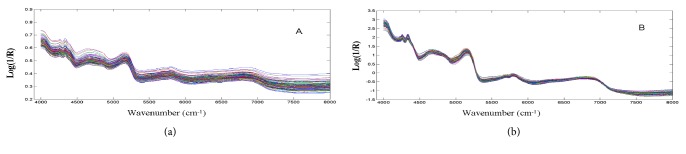
Raw NIR spectra of all samples and processed spectra.

Original NIR spectra of all samples are similar and broad; they consist of many overlapping narrow bands of different vibrational modes, as showed in Figure 1(a), the raw NIR spectra of 113 samples. It can be seen that the intensive spectral peaks are mainly in the region of 4000-8000 cm^−1^. The multiplicative scattering correction processed spectra of all samples (Figure 1(b)) showed the most intensive band in the spectrum belonging to the vibration of the second overtone of the carbonyl group (5352 cm-1); these are caused by the stretch or deformation vibration of C-H, O-H, and N-H groups, the first two of which are abundant in the flavonoids. Also, it might be caused by the combination of stretching and deformation of the O-H group in water for the spectral peaks intense absorption bands at 6900 cm^−1^ and 5180 cm^−1^.

### 3.3. NIR Calibration Model Establish

In this section, we aimed to establish a reliable and accurate calibration model for TFC and their antioxidant activities quantitative estimation. Thus, some process algorithms were taken into account. The whole calibration model established process in this study contains these steps.

**Firstly, **the PCA method was adopted to analyze these data for exposing cluster trends in the samples information.** Secondly,** some anomalous spectra were detected by using the Mahalanobis distance and hat matrix method to detect the outliers.** Thirdly**, after removing these anomalous spectra, the remaining samples were divided into a calibration set and a prediction set by using the Kennard-Stone (K-S) algorithm. The calibration set was used to optimize the model pretreat processes and establish the calibration model; the prediction set was used as external set to validate model. In the calibration model establish process, some spectra of pretreat methods and variable selective approach were optimized.

### 3.4. Principle Component Analysis

**Figure 2:**
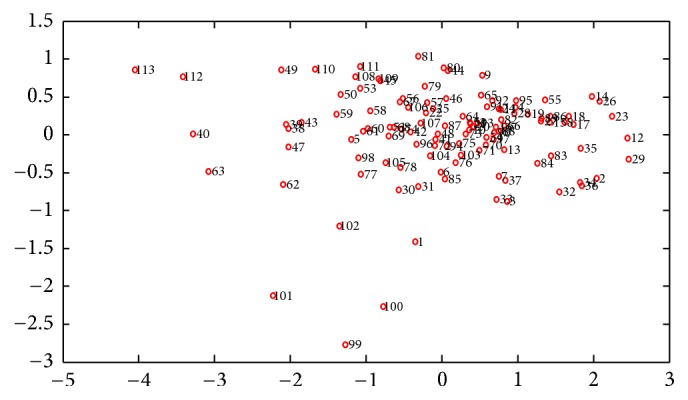
Investigation of dataset cluster trends by using PCA scores.

Principle component analysis (PCA) can be used as a preprocessing step to show samples distribution and visually descript the samples similarities and dissimilarities. In this section, we planned to use PCA to check the cluster of leaves' NIR spectra. Plotting PCA scores in two or three dimensions provides an effortless way to observe the data distribution. In the PC1-PC2 plot, the first two PCs contain about 95% (PC1: 90.32%, PC2: 5.17%) information of the raw data, and samples are unevenly distributed without obvious cluster which can be found (Figure 2). All samples were located at the whole positions of PCA scores plot. However, we still could find some samples, such as 99, 100, and 113 which are located far from other samples. These samples might be the outlier samples, which can affect the model calibration.

### 3.5. Anomalous Spectra Detection

**Figure 3:**
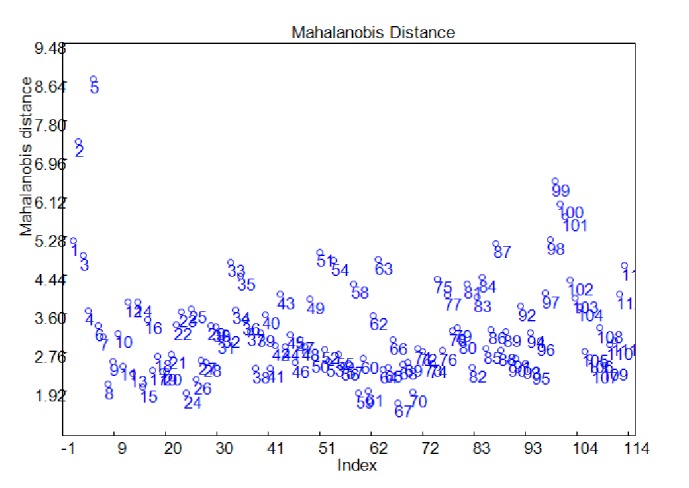
Mahalanobis distance analysis of all samples.

In this section, two outlier measure methods were applied to accurately explore the sample information. Techniques based on the Mahalanobis distance (MD) [27] and hat matrix [28] were applied in different fields of chemometrics such as multivariate calibration, pattern recognition, and process control. In the original variable space, the MD considered the correlation in the data, since it is calculated using the inverse of the variance-covariance matrix of the data set. The “Mahalanobis distance” between all the pairs of samples was calculated. As can be seen from Figure 3, 4 samples (2, 5, 99, and 100) were defined as the outliers as the four samples were significant far away other samples.

**Figure 4:**
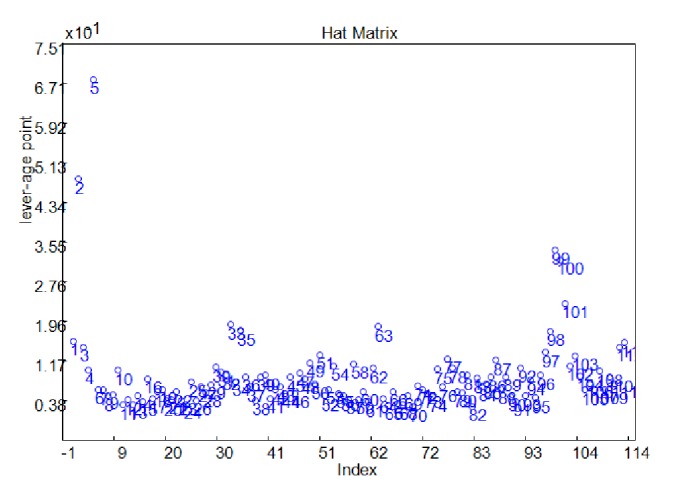
Hat matrix method analysis of all samples.

Furthermore, the hat matrix method was used to estimate the similarities of these samples. We can find that the 4 samples (2, 5, 99, and 100) were also chosen as the outliers (Figure 4). Based on these analysis, the four samples were deleted, and the remaining 108 samples were used for establishing the calibration model.

### 3.6. Signal Pretreatment and Prediction Model Establish

Kennard-Stone (K-S) algorithm was used to split the dataset into calibration dataset and prediction dataset with split ratio 80%. Thus, 88 samples were used to optimize the calibration model, and remaining 20 samples were used to estimate the established model. In the application of PLS algorithm, it is generally known that the spectral preprocessing methods and the number of PLS factors are critical parameters. Here, their effects on the results are discussed. The optimum number of factors is determined by the lowest root mean square error cross-validation (RMSECV).

**Figure 5:**
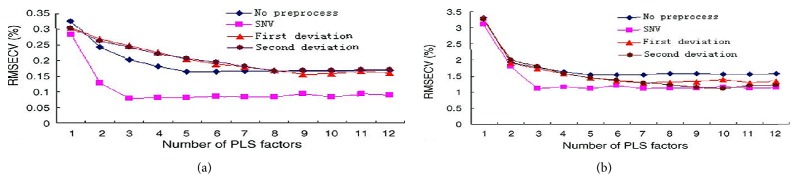
Optimized the best spectral process methods and number of PLS factors.

Pretreating spectra are a procedure to optimize data and avoid disturbance due to a changing baseline. Common used pretreatments method is averaging, smoothing and normalizing with first and second derivative spectra. The first derivative can eliminate shift errors and the second derivative eliminate tilt errors. Other methods such as multiplicative scatter correction (MSC), Savizaky-Golay method (SG), and standard normal variate (SNV) are also widely used in the NIR spectra. The number of PLS factors included in the model is chosen according to the lowest RMSECV. For RMSECV, a 10-fold cross-validation was performed. Figures 5(a) and 5(b) showed RMSECV plotted versus relevant PLS factors for determining TFC and their antioxidant activity with different spectral preprocessing methods, respectively. Standard normal variate spectral preprocessing method is obviously superior to other methods with lowest RMSECV values. Therefore, standard normal variate (SNV) spectral preprocessing method and corresponding optimized factor were selected to establish the calibration model.

### 3.7. Calibration Model Validation

**Figure 6:**
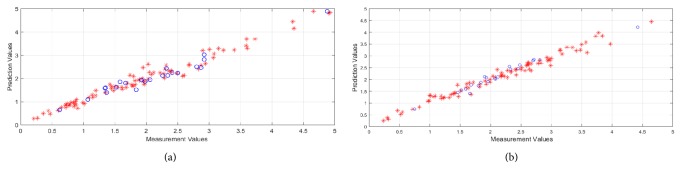
The square of correlation coefficient (R^2^) and root mean square error of prediction (RMSEP) for TFC (a) and total antioxidant activity (b).

The robustness of the method obtained by NIR technology was validated with the 20 prediction samples. The performance of the final PLS model was evaluated in terms of root mean square error of cross-validation (RMSECV), the root mean square error of prediction (RMSEP), and the square of correlation coefficient (R^2^). As can be seen from Figure 6, the coefficients of determination (*R*^2^) for TFC and total antioxidant activity prediction were 0.8863 and 0.8486, respectively, and the root mean square errors of prediction (RMSEP) were 2.203 mg g^−1^ and 0.2211 mM g^−1^, respectively.

## 4. Conclusion

In this study, a method was proposed to quantitatively analyze the TFC and their antioxidant activity of* Ginkgo biloba *L. by combining NIR spectroscopy coupled with chemometrics methods. The results verified that NIR spectroscopy was a suitable tool for quantification of TFC and their antioxidant activity, simultaneously. Comparing with other analysis methods, the NIR method has its advantages such as being simply pretreated, fast analysis speed, and being nondestructive; these make this approach has the potential of high sample throughput analysis and low costs and widely applied to products' quality control.

## Figures and Tables

**Table 1:** The total flavonoids content (TFC) and total antioxidant activity (TAA) of *Ginkgo biloba *leaves.

Samples	TFC^a^	TAA^b^	Samples	TFC^a^	TAA^b^	Samples	TFC^a^	TAA^b^
1	2.10 ± 0.18	0.47 ± 0.03	40	23.59 ± 1.79	2.98 ± 0.19	79	7.22 ± 0.46	1.01 ± 0.07
2	7.35 ± 0.42	0.99 ± 0.05	41	7.97 ± 0.48	1.06 ± 0.06	80	15.59 ± 0.95	1.96 ± 0.16
3	10.16 ± 0.76	1.93 ± 0.12	42	10.72 ± 0.98	1.14 ± 0.08	81	7.66 ± 0.68	1.64 ± 0.12
4	13.53 ± 0.87	2.12 ± 0.16	43	43.33 ± 3.16	3.97 ± 0.21	82	13.59 ± 0.97	1.93 ± 0.13
5	46.57 ± 3.25	3.48 ± 0.27	44	9.91 ± 0.69	1.19 ± 0.09	83	21.46 ± 1.46	2.94 ± 0.21
6	15.15 ± 0.93	2.33 ± 0.13	45	9.28 ± 0.64	1.75 ± 0.12	84	9.10 ± 0.54	0.83 ± 0.06
7	21.96 ± 1.98	2.67 ± 0.15	46	8.60 ± 0.63	1.00 ± 0.09	85	7.16 ± 0.58	0.57 ± 0.03
8	13.41 ± 1.15	1.52 ± 0.10	47	49.13 ± 2.67	3.84 ± 0.24	86	16.78 ± 1.07	1.24 ± 0.10
9	7.16 ± 0.63	1.51 ± 0.11	48	14.53 ± 0.93	1.99 ± 0.11	87	16.09 ± 0.99	1.39 ± 0.09
10	29.21 ± 1.92	2.45 ± 0.15	49	14.15 ± 0.96	1.47 ± 0.08	88	24.09 ± 1.54	2.81 ± 0.18
11	19.84 ± 1.82	2.34 ± 0.14	50	19.4 ± 1.65	2.51 ± 0.16	89	18.40 ± 1.48	1.68 ± 0.12
12	25.96 ± 2.29	2.83 ± 0.18	51	17.4 ± 1.34	1.68 ± 0.10	90	20.71 ± 1.35	2.79 ± 0.22
13	26.84 ± 2.32	2.59 ± 0.19	52	23.77 ± 1.87	2.69 ± 0.19	91	19.65 ± 1.28	2.62 ± 0.21
14	19.53 ± 1.75	1.82 ± 0.12	53	17.65 ± 0.95	1.99 ± 0.12	92	18.71 ± 1.23	1.66 ± 0.11
15	20.46 ± 1.69	2.08 ± 0.18	54	11.47 ± 0.73	1.19 ± 0.09	93	18.21 ± 1.01	2.23 ± 0.15
16	33.96 ± 2.98	3.26 ± 0.26	55	30.77 ± 1.98	3.15 ± 0.23	94	18.90 ± 1.42	2.18 ± 0.18
17	29.02 ± 1.92	2.33 ± 0.16	56	30.71 ± 1.85	2.84 ± 0.22	95	16.15 ± 0.97	1.60 ± 0.12
16	48.82 ± 3.44	4.42 ± 0.32	57	35.89 ± 2.45	3.18 ± 0.25	96	18.9 ± 1.12	2.78 ± 0.15
19	31.52 ± 2.76	2.60 ± 0.16	58	36.14 ± 2.24	3.50 ± 0.18	97	17.9 ± 1.53	2.91 ± 0.19
20	22.90 ± 1.54	2.04 ± 0.18	59	43.45 ± 2.89	3.57 ± 0.27	98	11.97 ± 0.98	1.22 ± 0.08
21	24.90 ± 1.59	2.67 ± 0.21	60	14.22 ± 1.07	1.47 ± 0.09	99	4.41 ± 0.35	0.72 ± 0.06
22	29.40 ± 1.97	2.96 ± 0.13	61	22.96 ± 1.48	2.64 ± 0.14	100	2.72 ± 0.17	0.32 ± 0.22
23	17.90 ± 0.98	1.61 ± 0.09	62	36.02 ± 1.91	3.59 ± 0.23	101	3.60 ± 0.23	0.52 ± 0.03
24	21.84 ± 1.89	2.57 ± 0.19	63	21.15 ± 1.58	2.62 ± 0.19	102	6.03 ± 0.51	0.84 ± 0.06
25	49.20 ± 2.92	4.65 ± 0.25	64	37.33 ± 2.68	3.78 ± 0.27	103	8.53 ± 0.64	1.40 ± 0.08
26	11.41 ± 0.70	1.19 ± 0.09	65	19.53 ± 1.39	1.72 ± 0.11	104	4.72 ± 0.36	0.55 ± 0.04
27	19.21 ± 1.06	1.80 ± 0.12	66	3.79 ± 0.27	0.71 ± 0.05	105	7.78 ± 0.42	1.35 ± 0.08
28	22.84 ± 1.42	2.93 ± 0.15	67	25.02 ± 1.52	2.07 ± 0.13	106	1.85 ± 0.12	0.97 ± 0.09
29	20.78 ± 1.39	2.50 ± 0.16	68	28.65 ± 1.86	2.31 ± 0.16	107	7.16 ± 0.58	1.91 ± 0.11
30	28.71 ± 2.35	3.41 ± 0.21	69	20.34 ± 1.53	2.17 ± 0.18	108	8.72 ± 0.63	2.41 ± 0.15
31	16.97 ± 1.04	1.65 ± 0.09	70	28.33 ± 1.94	2.19 ± 0.17	109	6.16 ± 0.51	0.55 ± 0.03
32	14.97 ± 0.78	1.32 ± 0.09	71	25.77 ± 2.05	2.42 ± 0.21	110	1.47 ± 0.93	0.24 ± 0.02
33	26.77 ± 1.85	2.99 ± 0.17	72	20.53 ± 1.86	2.18 ± 0.19	111	14.78 ± 0.54	2.81 ± 0.18
34	7.10 ± 0.55	0.81 ± 0.06	73	6.41 ± 0.51	0.88 ± 0.05	112	8.91 ± 0.61	1.42 ± 0.09
35	18.15 ± 1.07	2.63 ± 0.17	74	15.53 ± 1.27	1.46 ± 0.09	113	11.97 ± 0.85	2.42 ± 0.15
36	14.53 ± 0.96	1.39 ± 0.10	75	22.15 ± 1.75	2.28 ± 0.16			
37	29.02 ± 2.14	3.73 ± 0.21	76	8.35 ± 0.61	1.09 ± 0.07			
38	15.97 ± 0.98	1.28 ± 0.09	77	30.08 ± 1.98	3.00 ± 0.19			
39	24.02 ± 1.40	2.11 ± 0.11	78	32.27 ± 2.21	3.35 ± 0.21			

a: equivalent to rutin per g dry weight (mg g^−1^); b: equivalent to Trolox per g of dry weight (mM g^−1^).

## Data Availability

The data used to support the findings of this study are available from the corresponding author upon request.
